# Family planning decisions, perceptions and gender dynamics among couples in Mwanza, Tanzania: a qualitative study

**DOI:** 10.1186/1471-2458-13-523

**Published:** 2013-05-30

**Authors:** Idda Mosha, Ruerd Ruben, Deodatus Kakoko

**Affiliations:** 1 School of Public Health and Social Sciences, Behavioural Sciences Department, Muhimbili University of Health and Allied Sciences, P.O. Box 65015, Dar es Salaam, Tanzania; 2 Centre for International Development Issues Nijmegen (CIDIN) University of Radboad, Th.v. Aquinostraat 4 Postbus 9104, Nijmegen 6500 HE, the Netherlands

**Keywords:** Family planning, Decisions making, Perceptions, Gender dynamics

## Abstract

**Background:**

Contraceptive use is low in developing countries which are still largely driven by male dominated culture and patriarchal values. This study explored family planning (FP) decisions, perceptions and gender dynamics among couples in Mwanza region of Tanzania.

**Methods:**

Twelve focus group discussions and six in-depth interviews were used to collect information from married or cohabiting males and females aged 18–49. The participants were purposively selected. Qualitative methods were used to explore family planning decisions, perceptions and gender dynamics among couples. A guide with questions related to family planning perceptions, decisions and gender dynamics was used. The discussions and interviews were tape-recorded, transcribed verbatim and analyzed manually and subjected to content analysis.

**Results:**

Four themes emerged during the study. First, “risks and costs” which refer to the side effects of FP methods and the treatment of side -effects as well as the costs inherit in being labeled as an unfaithful spouse. Second, “male involvement” as men showed little interest in participating in family planning issues. However, the same men were mentioned as key decision-makers even on the number of children a couple should have and the child spacing of these children. Third, “gender relations and communication” as participants indicated that few women participated in decision-making on family planning and the number of children to have. Fourth, “urban–rural differences”, life in rural favoring having more children than urban areas therefore, the value of children depended on the place of residence.

**Conclusion:**

Family Planning programs should adapt the promotion of communication as well as joint decision-making on FP among couples as a strategy aimed at enhancing FP use.

## Background

Many sub-Saharan Africa countries have high rates of unmet need for family planning (FP) [1,2] and low rates of contraceptive use [2]. Individuals and couples who want to limit their fertility, are often unable to obtain the FP methods they need due to numerous barriers [3]. These barriers include high cost, long distances, poor distribution, medical restrictions and fear of side-effects, or even misinformation. The lack of understanding surrounding what influences FP use and how decision-making takes place in families has lead to the inability of policy and programs to focus on the factors that are most important to helping people control their fertility [3]. Although much of the available literature assumes that financial cost is the primary factor inhibiting contraceptive use, various studies around the world suggest that fear of side - effects of FP are more influential in decision-making [3-7]. It is estimated that 59% of unintended pregnancies could be eliminated if method-related reasons for non-use were overcome; and fear of side-effects is the most commonly cited reason for such non-use [4]. For instance, in the Colombia 2005 Demographic and Health Survey (DHS) [5], 21% of married women with unmet need for FP cited health problems or side-effects as their reason for non-use, while 10% cited cost/access and none cited lack of knowledge. Also the 2001 Uganda DHS [6] found that 25% of married female non-users cited health/side-effects as the reason for non-use, 20% cited cost/access, and 5% reported lack of knowledge. Similar trends were established in Asia, South America and Africa [7].

Fear of side-effects is also a commonly cited reason for contraceptive discontinuation [8]. Many studies have found that while some of these are based on actual health related side-effects, many fears are based on rumors, rather than personal experience [3,9-14]. A study in Nepal found that side effects were the main reasons cited for discontinuing the use of FP and that most people received information about FP from mass media [15]. In Nigeria, knowledge of FP is generally high; however, use remains low. The main reasons for this lack of FP use include fear of complications, lack of understanding of methods and fear of opposition from the husband [16]. Nadia et al. [17] identified evidence of fear of FP side-effects among females and males from India, Nepal and Nigeria. Furthermore, fear of side-effects from hormonal methods among male partners has also been found to impact females FP decision-making and their fear to use FP [18]. Generally, researches show that spousal communication can increase contraceptive uptake and continuation [19-22]. Moreover, it is clear that spousal discussion and partner approval are significant in inducing a woman to use modern contraceptives in the Central Terai region of Nepal [23].

Determinants of spousal communication are varied and complex. In Sub-Saharan Africa, gender roles and norms are particularly salient, shaping spousal communication and subsequent FP decision-making in significant ways. Although contraceptive methods and services are frequently geared toward women, men are often the primary decision-makers on family size and their partner’s use of FP methods [20,24,25]. In addition, spousal disagreement can serve as deterrent because women might fear initiating a difficult conversation about FP [26]. On top of that, evidence suggests that communication between couples may influence FP method choice and frequency of use among women already using contraception [27-29]. Despite the clear association between spousal communication and contraceptive use, little is known about how communication dynamics influence FP decision-making. For example, what is the content and pattern of decision-making around contraceptive use among couples, and how do women and men perceive this process in the context of their relationship? [12,30,31].

Opposition from male partners has been cited as an important factor that affects FP use [32]. In Ghana for example, ancestral customs give men rights over women’s procreative power [33]. In fact women in poorer countries with lower levels of education show the highest rates of unmet needs for FP [34]. In addition, men have traditionally been portrayed as either explicitly or implicitly unconcerned or unknowledgeable about reproductive health. Generally, men have been regarded as formidable barriers to women’s decision-making about fertility, contraceptive use and health care utilization [35].

Women’s participation in domestic decision-making is increasingly being recognized as affecting their ability to make reproductive decisions. Demographic literature suggests that active involvement in domestic decision-making indicates the power of women within the household and, consequently, their ability to control their fertility [36,37]. Several studies have found that woman with little autonomy in the household are less likely to make innovative decisions [11,31]. The influence of gender-based power dynamics in sexual relationship between men and women on reproductive outcomes is becoming increasingly recognized [38,39]. The empowerment of women as reflected in their socio-economic and employment status, educational levels, household organization, the dynamics of their marital relations and their involvement in domestic decision-making is an important factor in the decline of fertility levels in developing countries. This connection between paid employment and demographic behavior has been found to be strong, particularly in its impact on contraception and fertility [31]. The rationale behind this connection is that the financial contribution to the household by women with paid employment is higher, hence enabling them to control resources and household expenditures, as well as their reproduction [40].

As FP programs challenge complex societal norms, they may also challenge traditional gender roles and dynamics and reshape social norms, for example, by endorsing women’s right to refuse sex, and by encouraging couples to discuss and jointly decide on an appropriate contraceptive method [41].

In Tanzania at 5.4 the Total Fertility Rate (TFR) is still high. Currently, 34% of married women in Tanzania use some method of contraception. Of these, 27% use a modern method and 7% use traditional methods. The most commonly used methods among married women in Tanzania are injectables, pills and female sterilization [42].

Between 1991 and 2010, five nationally-representative surveys have measured contraceptive use among currently married women in Tanzania. The surveys show that during the last 19 years, there has been a gradual but steady increase of contraceptive use among currently married women, from 10% in the 1991–92 Tanzania Demographic Health Survey (TDHS) to 34% in 2010. In addition, the use of modern contraception methods increased by 20 percentage points, from 7% in 1991–92 to 27% in 2010. The Contraceptive Prevalence Rate (CPR) has increased from 26% of married women in 2004–05 to 34% in 2010. And the current use of modern contraceptive methods among all women has increased from 18% in 2004–05 to 24 percent in 2010 [42].

Despite the ready availability of FP methods and high contraceptive knowledge, the use of FP methods remains low. In this study, use of modern contraception refers to current use. It is not well-established how people make family decisions on FP use. Neither have their perceptions on FP been well established. These are important issues to be addressed so as to enhance further contraceptive use and lower fertility levels in the East African countries. This study therefore, sought to assess FP decisions, perceptions and gender dynamics among couples in Mwanza, Tanzania. Specifically, the objectives of this paper are threefold: first, to report about people’s perceptions of FP methods in Tanzania; second, to report on the people’s perceptions of FP methods use; and third to report about how gender dynamics impinge on FP decisions. The findings of the present study are expected to contribute insights on the potential interventions that could be designed to further promote the use of FP.

## Methods

### Study design and setting

This study employed a qualitative study design. It used focus group discussions (FGDs) and in depth interviews (IDIs) with men and women who resided in the study areas to generate the necessary information. The FGDs approach capitalizes on group dynamics in which interactions help to explore people’s understanding of the phenomenon under study as well as of the norm system influencing their perceptions [43]. In addition, this approach takes advantage of in-depth information from the participants on the study subject.

### Setting

Mwanza region has an area of 19,592 km^2^. Administratively, it is divided into eight districts, namely Ukerewe, Magu, Sengerema, Kwimba, Nyamagana, Geita, Misungwi and Ilemela. According to the TDHS [42], 15.2% of married women in Mwanza were using at least one method of contraception. Out of those, 11.7% were using modern contraception and 3.5% were using traditional FP methods [42]. Mwanza has relatively well-established and functioning public and private health facilities offering FP services. The region has a total of 377 health facilities, the majority of which offer FP services. The facilities in the region range from dispensaries, health centers, district hospitals, one regional hospital and a referral hospital. Furthermore, Mwanza is one of the regions in Tanzania with high unmet needs for contraceptives (21.6%) and has a high fertility rate of 5 [42]. The region has a population growth rate of 3.2% per annum [42]. All these factors made Mwanza an ideal region for executing this study. Field research for this was conducted between June and September 2010.

### Sampling

Three districts, namely Ilemela, Magu and Misungwi, were selected out of the eight districts available in Mwanza region for this study. The three districts represent urban, semi-urban and rural areas, respectively. From the districts, one ward was selected and from each ward one village was selected; and from the village one street/hamlet was selected as the study area. With help from local leaders, we employed purposive sampling to obtain 98 males and females discussants aged between 18 and 49 either married or cohabiting. A minimum number of 32 participants were selected from each district with an almost equal number of both sexes. This was done with the help of the local leaders of each respective area. We used purposive sampling to get people who could provide information that we needed. Purposive sampling for variation enabled us to hear different opinions on the study subject.

Furthermore, to supplement the information that we got from FGDs, six key informants one female and one male, married or cohabiting aged between 18 and 49, were purposely selected from each district to participate in the study with the help of local leaders. In all, three men and three women were interviewed as key informants in this study.

### Data collection methods

#### Focus group discussions (FGDs)

A total of 12 FGDs were conducted: four group discussions in each selected districts; two among females and two among males. The participants for the FGDs were purposively recruited with the help of local leaders. We conducted separate FGDs for male and female participants. To facilitate discussions among the study participants, we separated participants into youth and adults groups (i.e., 18–29 and 30–49 years) for both categories of females and males, see Table 1 below.

**Table 1:** Diagrammatic presentation of the sample size for FGDs and IDI

**Districts**	**Number of IDIs**	**Number of FGDs**	**Focus group participants**
Ilemela	2 a man and woman	4	32
Magu	2 a man and woman	4	32
Misungwi	2 a man and woman	4	38
**Total**	**6**	**12**	**98**

The FGD guide focused on participants’ perception of FP methods, use of FP, communication on FP and household decision-making on FP. The discussion guide was prepared in English and translated into Kiswahili by the principal investigator (PI). FGDs were held in Kiswahili, Tanzania’s national language and universally accessible. The PI moderated the discussions with the help of a research assistant, who took notes and kept time. Only one FGD was executed each day. Scheduling allowed for reflection and consolidation of emerging issues for further interrogation. Each session lasted, on average, one and-a-half hour.

#### In-depth interviews (IDIs)

Six IDIs were conducted to supplement the information generated from the use of FGDs. The informants were married or cohabiting males and females aged 18–49. Informants were purposefully selected with the help of local leaders. One male and one female were selected from each district. The interview guide was originally prepared in English before being translated into Kiswahili by the PI. The PI conducted in-depth interviews in Kiswahili, a language in which all the informants were competent. Each interview session lasted half an hour. One IDI was executed each day. This facilitated reflection on, and consolidation of, emerging issues for further questioning. The interview guide focused on FP decisions, couples perception of FP, and association of FP with marital infidelity. Conducive places were secured to provide privacy and free conversation between the PI and the informants. All FGDs and IDIs were tape recorded, after seeking consent from the participants. See Table 1 below.

#### Data analysis

The tape-recorded discussions and interviews were transcribed and translated into English and thereafter back translated into Kiswahili. The first author supervised transcriptions from tapes and translations into English. Content analysis was carried out following the guidelines by Graneheim and Lundman [44]. The first author analyzed the data manually by initiating the coding and category assignments. Then the second and third authors went through the data identifying discrepancies. The discrepancies were discussed and consensus was reached after referring to the tapes. Codes and categories that emerged from data were later sorted out to form the main themes that emerged, as presented in the findings section.

#### Ethical considerations

We obtained a research permit both from the Commission for Science and Technology Tanzania (COSTECH) and ethical clearance from Muhimbili University of Health and Allied Sciences (MUHAS) Institutional Review Board, the two authorities with such powers in the country. We also obtained permission to conduct the study from the regional, district, wards and village authorities. Individual verbal consent was sought and obtained from the study participants prior to their participation in the FGDs and in-depth interviews. All information was kept confidential, with names excluded from the recorded materials to avoid giving away the identity of the participants.

## Results

We present the findings from FGDs involving 98 discussants: forty eight (48) males and fifty (50) females. Also, we present the findings from the six IDIs: three from females and three from males. The mean age of the study participants was 36.5. The main themes that emerged are as presented below.

### Risks/costs

Use of FP was generally associated with marital infidelity. Some men worried that the methods women used allowed them to have extra-marital affairs without being discovered by their partners, since they would not be able to conceive. One man commented:

*When a woman starts proposing FP use, or asks about the number of children we should have, the first thing I ask her is whether she intends to cheat or not?* (FGD, males, rural, 30–49 years).

Another man in the semi urban area, who insisted that women using FP tended to have extra-marital affairs, gave similar sentiments.

*Some women can have affairs with other men if they use FP methods because they will not conceive…* (FGD, males, semi-urban, 18–29 years).

Such sentiments were shared by some of the women, who said that the use of FP was a sign of faithlessness. One woman commented:

… *If you try to discuss FP, or you want to use FP, then he will ask you, what do you lack? What do you want to do? Why do you want to use FP? Some men say that if you use FP you will be unfaithful because you will not become pregnant when you cheat on him…*(FGD, females, rural, 18–29 years).

However, the in-depth interview informants had different views. Some of the informants said that women could use FP methods, after some discussion and agreeing on the issue with their husbands. If the woman does not discuss and agree on the use of FP with their husbands, then husbands suspect the woman of having an illicit affair. Although some of them agreed that secretive use of FP among women could make someone suspicious, discussants generally affirmed the use of FP. One man opined;

Long time ago people linked the use of FP with infidelity, nowadays people know the importance of using FP, and see it as a normal thing, it is not like in the past when people linked FP with infidelity….(IDI, male, rural, 45 years ).

### Risks/costs: perceptions of FP methods side-effects

In the FGD, males and females were both concerned about the side-effects which they feared could occur due to the use of FP methods. The extreme “side effects” mentioned are the result of myths/misconceptions within the community. Males were afraid to allow their wives to use FP methods because they had heard that FP methods have side-effects for women. On the one hand, they mentioned minor side-effects such as headaches, bleeding, weight gain, weight loss, nausea, dizziness and stomach-ache. On the other hand, they pointed out severe side effects such as infertility, cancer and birth deformities including physical and mental handicaps. For example, one male commented:

…*Women have irregular periods after using contraception. In addition, once a woman uses contraception later on can give birth to a mentally-retarded child or one with missing organs such as eyes or arms… the woman can give birth to a child who looks like an animal or like a goat* (FGD, males, rural, 30–49 years).

Another man had this to say:

*These FP methods spell trouble for women and our families. For example, if an implant is inserted into a woman’s arm, then she is told not to do hard work such as farming. I wonder how that woman will survive when she earns her food through farming…. It is better that women should not use FP methods because it will make our lives poor* (FGD, males, semi-urban, 30–49 years).

Similar sentiments on the side-effects of FP were pointed out in another FGD. One man said:

*These FP methods have side-effects on our wives… some of women suffer from uterine tumours, cancer and irregular periods when they use FP methods* (FGD, males, semi-urban, 18–30 years).

Women also raised concerns over what they saw as probable side-effects of using FP methods, including cancer, over-bleeding, uterine tumours and infertility. For example, a female discussant said:

*FP methods have some side-effects to women. I used FP injection for about three years. I started over-bleeding for three months…I was so scared… I stopped using them. Also, one of my friends who was using injections (Depo-Provera) suffered from uterine tumours. I also heard that FP methods can cause cancer* (FGD, females, rural, 18–30 years).

Similarly from the IDIs, informants said that they feared the side-effects caused by some of the FP methods. As one explained:

*I used oral pills and started getting my periods irregularly. I went to the hospital and changed into an implant. I suffered from heavy periods and headache… Again, I went to the hospital and opted for an injection. Since then, I have no problem. ..(*IDI, female, urban, 34 years).

Another woman commented:

*The majority of women fear to use FP methods because of the side-effects they face. It is scary. I think people need more information on FP side-effects and what they should do when they have those side effects* (IDI, female, rural, 40 years).

Some of the men mistakenly believed that long-acting contraceptive implants could travel throughout the body and get lost, and causing harm to the users.

Also, some participants showed general lack of knowledge and information on FP methods, as one woman pointed out:

*The majority of males and females do not use FP because of lack of knowledge and information about FP side-effects and how to overcome them. Some, people especially men, do not have enough information on FP…(FGD, females, rural, 18–29 years*).

### Risks/costs: financial consequences in relation to FP side effects

Men who perceived that FP had side-effects, were also concerned about the financial implications for treating their wives once they experienced FP side effects. In fact, many of these men, especially from rural areas, argued that they were poor and did not have enough money to pay for treatment in case their wives suffered from FP side- effects. Some men argued that the government should set aside money for treating women who experienced FP-related side-effects. They pointed out that they disapproved of their wives using FP methods because of financial repercussions from treating their wives if they experienced FP side effects. One man said:

*We cannot allow them (wives) to use FP because if they become sick we will not be able to pay for their medical treatment because we are poor…* (FGD, males, semi-urban, 18-29 years).

The women were also concerned about who would foot their medical bills if they suffered from side- effects due to use of FP. They argued that, as women, they depended on their husbands for medical treatment. One of them said:

*I am afraid of using FP methods. Who will pay for my medical treatment in case I suffered from FP side effects?…* (FGD, females, rural, 18–29 years).

Also, the IDIs informants raised concern over some side effects they associated with FP methods. For instance, one woman stated that:

*These modern FP methods have several side-effects to women. For instance, I used oral pills for one year. I started over-bleeding and having irregular periods. I went to the hospital and I was told to stop using them. I think if I didn’t go to the hospital I would have suffered a lot* (IDI, female, urban, 38 years).

Another informant said:

*My wife used Depo-Provera injection for 2 years. She stopped two years ago and we wanted to have another child. She is not able to conceive up to now. We have been consulting different doctors who keep on examining her and telling her that everything is okay. But how? We have spent a lot of money on bus fares and consulting these doctors. We are very worried that she will not be able to conceive another baby* (IDI, male, urban, 41 years).

### Gender relations: covert use of FP

Many women held the view that FP methods helped them to plan and space children and improve their general health situation. These women pointed out that since they spend most of their time with their children, they are the ones who see their children suffer from hunger and from other basic needs. As a result some women used FP methods without their husbands’ consent. This was done deliberately to protect their health and the plight of their children. One woman said:

*I have five children by caesarean section. I have been convincing my husband on using FP methods and he refuses. He wants more and more children. I almost died when I delivered my last child…. Having seen our condition at home that we don’t have enough food and basic necessities, I decided to undergo sterilization without my husband’s consent* (FGD, females, semi-urban, 30–49 years).

Some women were concerned about spacing children and their individual health, and, therefore, used FP methods even when they had heard of the family planning side effects. One woman stated:

I did not want to bear children so closely because my health would be jeopardized. I asked my husband that we use FP methods, but he refused, saying that FP methods could cause infertility.

*I decided to use FP injectables secretly and I have been doing so for two years now. I don’t want to see my children going hungry or turn into street children* (FGD, females, semi-urban, 18–29 years).

During the IDIs, some informants pointed out that some women used FP clandestinely as their husbands oppose the use of FP methods.

*…Some women can use FP without the consent of their husbands, because their husbands disapprove of FP use. But couples who approve of FP, discuss with their partners and reach a consensus on using FP* (IDI, male, urban, 39 years).

Other women feared that if they used FP methods and suffered side-effects, it would be easy for their husbands to discover that they were using FP behind their backs. In that case, they feared being divorced by their husbands.

One woman alleged:

*Some of us are afraid of using FP methods without our husbands’ consent. If we use FP methods and suffer from side-effects, our husbands will not pay for our medical treatment. We could be left untreated… to die … we could be divorced, because we have gone against our husbands’ wishes* (FGD, females, semi-urban, 18–29 years).

### Gender relations: couple communication

Communication among the couples is important in FP use and decision- making on the number of children a couple wants to have. In rural areas, there was little or no communication among the couples on the use of FP and on desired number of children. As one participant put it:

*In rural areas many couples do not discuss FP… the majority of them lack FP knowledge …* (FGD, males, urban, 30–49 years).

In addition, the findings show that some people believe that discussing FP issues with their partners was not that important. For example, one woman had this to share:

*Most people in rural areas do not discuss FP issues with their partners. Some men believe that it is not an important thing to them. Other men believe that it is the responsibility of women that is why they don’t discuss it* (FGD, female, rural, 30–49 years).

Another man put it in this way:

*I think it is not important to discuss FP issues or the number of children to have with your partner. Children are a blessing from God, and knows what they will eat. That is why even in the Bible, family planning is not mentioned* (FGD, male, 18–29, urban)*.*

Moreover, the findings show that it was difficult for women to engineer discussions, as they perceived that men largely made key family decisions. One woman commented:

*Men are the decision-makers in the households, including on the number of children to have and FP use or not use….* (FGD, females, rural, 18–29 years).

In urban areas, participants expressed different perceptions, sharing that most couples talk about FP and number of children to have. One woman said:

*In urban areas, most couples discuss about FP and number of children to have… life in urban is hard people want to have children who they can feed and take care of* (FGD, females, urban,18-29 years).

Likewise, the findings from the IDIs show that most of the couples nowadays discuss FP use because of economic hardship. People want to have manageable families they can afford to take care of. As one key informant said:

*It’s normal nowadays for couples to discuss FP. Life nowadays is hard and people want to have the right number of children they can take care of. People do not want to have children who will turn into street-children* … (IDI, male, urban, 39 years).

Another woman opined:

*I discuss the number of children to have as well as the spacing between our children with my husband. We must prepare ourselves before we add another child. We must plan our lives beforehand* (IDI, female, urban, 29 years).

### Male involvement

Findings show that participants were of the view that, traditionally, men were the heads of households and decision-makers in all issues in their respective households. Men decide on FP and the number of children as well as how to use what is produced by the family. Also, the findings show that since men were the decision-makers, they were expected to initiate discussions on FP and the number of the children the couple want to have. Men were perceived as the sole providers for their family needs. Women were not considered decision-makers, but implementers of what had been decided by men, without questioning men’s decisions. As one male commented:

*In this place, men don’t discuss FP, because we think there is no need to…, men are the decision-makers. They can tell their wives that they should have ten children and that is it. It is, a man who has to decide. A woman cannot oppose anything that has been decided by a man* (FGD, males, rural, 30–49 years).

A rural woman made similar comment during the FGD:

*A man decides on FP and the number of children to have. If a woman decides the man will ask her whether she is the one who feeds the children? Or whether she is the one paying for school fees? ….* (FGD, females, rural, 18–29 years).

Similar sentiments were given in a semi-urban area. One woman states:

*Traditionally men have to decide on issues related to FP, although you can discuss them with your husband; however, he is the one with the final say* (FGD, females, semi-urban, 30–49 years).

Some women went against the norm, noting that women were the decision-makers. They pointed out that FP issues were in the women’s sphere and, thus, they should be left to decide on the number of children as well as the FP methods to use. After all, they argued women were the ones who suffer during pregnancy and delivery. That was why some women used FP methods without their husbands’ knowledge. One woman stated:

*I think women should decide on the number of children to have. For instance, I had eight children and was tired of giving birth to more children while we were poor. I asked my husband for permission to use FP methods but he refused. However, I started using FP methods secretly* (FGD, females, rural, 30–49 years).

On the other hand, men viewed themselves as the decision-makers in their households, arguing that they provided for the households’ needs and so they should be the decision-makers. Also, they contended that Sukuma traditions and customs recognize men as decision-makers. (Sukuma is an ethnic tribe in Tanzania).

For example, one man stated:

*Men are the decision-makers in all matters in the households. They are the head of the households and provide for the family needs. Even our traditions and culture recognize men as the decision-makers* (FGD, males, semi-urban, 30–49 years).

Another man explained:

*Women are not decision-makers… and have to move from their parents to their husbands’ homes. A man in African families is the one with the last decision….* (FGD, males, semi-urban, 18–29 years).

Indeed some of the discussants acknowledged men as the providers for the families. The men were perceived as heads of the households. Even in cases where women produced or owned some resources that contributed to the family livelihoods they remained invisible due to the dominant patriarchal norms that treat men as sole breadwinners. For example, one man said:

*Men are the bread-winners, they provide everything for their families, and women just stay home taking care of the children…* (FGD, males, rural, 18–29 years).

Another woman elaborated:

*We take care of the children and family. Sometimes, we work in farms but all that we produce belongs to our husbands. We cannot do anything without the consent of our husbands even with what we produce* (FGD, females, rural, 18–29 years).

Some of the participants underscored the importance of maintaining marriage in their society so as to take care of their children. Also, female participants alluded to the importance of respecting their husbands by informing them about FP use to maintain marriages. One woman said:

*I think it is important for all of us to protect our marriages. We should discuss and get the consent of our husbands before starting using FP methods. If we use FP without our husbands’ consent our marriages can break down and cause problems to our children* (FGD, females, semi-urban, 18–29 years).

Another woman in the rural area commented:

*I think women should not use FP methods without informing their husbands … using FP methods without informing partners can cause marriage break ups …* (FGD, females, rural, 18–29 years).

Interestingly, some of the participants mentioned households where women both produced and fed their children, while their husbands did nothing apart from drinking. These participants clarified that not all men are the bread- winners for their families; some are just ceremonial heads of households, while their wives handle all the households’ responsibilities. One female key informant said:

*Some women work hard to feed their families while their husbands drink everyday and they don’t care about their families* (FGD, females, semi-urban, 30–49 years).

During the in depth-interviews, some of the informants offered more nuanced views on male dominance. One key informant put it in this way:

*If you love your wife, you will discuss everything with her, because it takes two people to have a baby. I think couples are supposed to discuss with their partners on use or non- use of FP* (IDI, male, urban, 39 years).

### Urban/rural differences: value of children and use of FP

Particularly in the rural areas, men expressed preference for large families and perceived FP methods as tools for controlling the number of children, contrary to their preferences. Some women said that men wanted to have many children, and hence perceived men as reluctant to allow their wives to use FP methods. As one discussant explained:

*I think some men want more children than their wives For instance, I wanted four children but my husband wanted more…* (FGD, females, urban, 30–49 years).

Unlike in urban areas, people in the rural areas prefer many children because they help them with farming activities. In fact children in rural areas were generally treated as sources of labor for families. Social norms in rural areas favored having as many children as possible because of the extended family support system that allowed the children to stay with relatives. One woman commented;

*…Ten children can participate in farming activities and produce more than a person with two children* (FGD, females, rural, 18–29 years).

In addition, findings show that relatives had influence on the number of children a couple might have. Relatives, especially mother-in-laws, could put pressure on their sons or daughter-in-laws to have more children than they had initially planned to have. For example, one woman said:

*Sometimes we are afraid to discuss FP with our husbands because some mother-in-laws had made it clear that they want their sons to have as many children as possible*. *We are afraid to go against our mother-in-laws … for that matter, we do not discuss FP with our husbands* (FGD, females, urban, 18–29 years).

In urban areas, on the other hand, the value of children was seen in terms of costs involved in raising children, especially in terms of school, medical services, and other social amenities. Moreover, in the urban areas everything was paid in monetary terms ranging from renting houses, buying food and other amenities. Thus, having more children would mean incurring more costs.

One woman in the urban area commented thus:

*In this area, people plan their families and most people use FP methods because life is hard and expensive in urban areas. We buy everything that we eat because we don’t have farms. We use FP methods to have families that I can take care of. In the urban areas, most couples discuss FP and the number of children to have…* (FGD, females, urban, 18–29 years).

Similar views came out during the FGD with men as one of them said:

*Having many children in urban areas can create difficulties in getting their needs. For everything, you have to pay money; schools fees, medical services… With many children you will not be able to meet their needs* (FGD, males, urban, 30–49 years).

Furthermore, one key informant commented:

*Nowadays, people know the importance of using FP because life is hard. People want to have children whom they can take care of* (Male, 39 years, IDI Urban).

## Discussion

The study aimed to assess people’s perceptions of gender dynamics regarding FP use. The direct quotes of women and men on their perceptions of the FP methods have been presented to allow the reader to ascertain the validity and dependability of the study findings. In fact, peoples’ perceptions of FP methods in this study are similar to people’s perceptions on FP in other countries in sub-Saharan Africa [17,29,45-48]. From the study, four main themes emerge: risks and costs; male involvement; gender relations and communication; and urban–rural differences.

### Risks and costs related to FP

Concerns about the side-effects of hormonal contraceptives and its consequences from this study are sub-divided into the following parts:

a) Social risks

Study findings reveal that FP was not only about child spacing and birth control, but also about the social, economic and cultural aspects of the society. In this study, perceptions on different kinds of risks came forward as relevant to FP, and as having an influence on FP use and communication among couples in the households. The anticipated side effects, as depicted in this study, imply that financial costs would be involved in treating perceived side effects. These side-effects worried some women, especially those who are too poor to afford to pay for the medical treatment. Generally, these women relied on their husbands to pay for their medical treatments, as they did not have their own money.

Furthermore, women also considered the social risks of being labeled as unfaithful by using FP methods. Fear of social risks could lead to low or non-use of FP among women and therefore, prevented women from discussing FP with their husbands. This finding is in line with Kaida et al. [45] in Uganda, where contraceptive use was commonly associated with promiscuity and infidelity. Also in Ghana, contraceptive use was associated with promiscuity [46]. In Ghana, sixty percent of women agreed with the statement that women who use contraception may become promiscuous [46]. These different kinds of risks on FP were gendered in the sense that men and women spoke of different kinds of risks. Men worried about the financial costs and health risks to their wives, as well as the health status of their children. Women, on the other hand, were worried about side effects and the risk of being accused of promiscuity. This mismatch or disconnect on FP affected the couples’ FP communication. As a result, there was little or no communication between husbands and wives on FP issues, which in turn lead to low or non-use of FP methods. This lack of joint decision-making among couples on FP corroborates with the study by Mwageni et al. [49], which found lack of joint decision-making on family planning issues among the couples in region Mbeya, Tanzania.

b) Health risks

Some of the respondents pointed out health risks they associate with FP methods, including having deformed and mentally-retarded children. This fear caused many people to shun using FP methods. These findings corroborate with other studies on FP [3,9-14,45,47] where it was established that the fear of side-effects was widespread.

In our study, the primary reason given by women for not using hormonal methods such as oral pills, intra-uterine devices (IUDs,) injectables and implants was fear of perceived side effects. Women were afraid of FP side-effects such as heart palpitations, irregular menstrual cycles, dizziness, and weight gain or loss; they also perceived IUDs and implants as painful. This outcome corroborates other study findings [48; 3, 9–14] which gathered the same complaints of FP methods from the study participants.

In addition, two studies conducted in Pakistan show that fear of side effects was also found to be one of the most common explanations for non-use of contraceptives [50]. In Mali, some women were concerned that oral contraceptives and injectables could cause permanent infertility [10].

### Gender relations and communication among couples

Gender relations are pervasive, but also dynamic and change over time. Gender inequality in reproductive decision-making is a key element of the social context of reproductive health. Researches show that couples often disagree about the desirability of pregnancy and the use of contraceptives [50,51]. When this discordance occurs in a situation of male authority, men's opinions about these issues may overrule women's, even though the women often must implement the decisions made on these matters. In some cases, husbands fear that if they approve of FP and allow their wives to use it, they would lose their role as heads of their families or their wives may be unfaithful or they might lose face in their community [11].

In our study, we found covert use of FP that could have been caused by inequality of gender relations among couples which led to lack of communication between them. However, people, especially males, associated covert use of FP with infidelity among the females. This corroborates another study by Population Council 2001 [52], which found that women may practice contraception covertly, potentially exposing themselves to financial vulnerability or emotional or physical violence if discovered [52]. It also corroborates a study by Jessica et al. [51), which found that many wives acted independently and often contrary to their husbands’ desire through their covert use or non-use of contraception and pregnancy termination. Also the findings are in the same line with a study in Malawi where women were found to use FP secretly [51]. Other researchers noted that cultural and contextual factors may impede the discussion of family planning and contraception, especially among younger couples [53,54]. In this situation the discussion of sex with the opposite sex is impossible, especially when cultural taboos are factored in. Furthermore, in our study males argued that the use of FP methods among females could enable them to have affairs with other men without being discovered by their husbands.

The findings of this study revealed some changes in gender relations, particularly when it comes to decision-making in the households among men and women on the use of FP and the number of children to have. Participants talked about communication among husbands and wives on FP decision-making in the households and wives on FP decision-making, especially in the urban areas, where some women reported discussing FP with their husbands as compared to rural areas where women could not introduce FP issues before their partners. In addition, women in urban areas expressed freedom to initiate a discussion on FP without fear of being labeled as unfaithful wives. This could be caused by information and education, both more readily available in urban areas as compared to rural areas. The high cost of living in urban areas made couples think twice about the costs of bringing a child into the world. In rural areas on the other hand, children are perceived as assets, and sometimes work on farms hence the more children one has the better.

Furthermore, this study found that there was no communication among the couples, especially in rural areas about FP and, to some extent, this contributes to covert use of FP. The absence of communication might have been caused by culture prevailing stereotypes that only men should initiate the discussion of FP since they are the bread- winners and pay the bride prices when they marry. In rural areas, lack of communication could be attributed to the dictates of patriarchy, where living in a male-dominated society makes such discussion out of bounds for some couples [55]. It was shown by Khan et al. [55] that FP was the least discussed in their study. This corroborates a study done by Blanc [56], which shows that “verbal communication between partners about reproductive health is low in many developing countries and that gender-based power inequities contribute to a lack of communication.” Indeed, the idea of not communicating with one’s spouse about FP in rural areas seemed normative and is reflected in a statement by one of the discussants who participate in our study: ‘communication *about family planning issues is of no importance.”*

Furthermore, inequality in gender relations among the couples can lead to poor health outcomes by hindering communication between partners about reproductive health decisions; by constraining women's access to reproductive health services, by preventing women’s and men’s attainment of sexual health and pleasure; and by increasing their risk of contracting HIV infection and other STIs [57].

### Male involvement

Male involvement in reproductive health issues is of paramount importance in many societies, where they hold decision- making power in the household. However, in our study men showed little interest in participating in FP issues, in addition to little knowledge on FP methods. The findings also show that men were reluctant to escort their expectant wives to antenatal clinics. Men consider it to be women’s to attend antenatal clinics. It could be argued that men are also interested in FP, although information is more typically and easily conveyed to women rather than men. This could have been caused by previous FP programs in Tanzania that targeted women and children and, thus, contributed to men’s alienation from FP. An example of previous FP programs in Tanzania is Maternal and Newborn Child Health (MNHC). In consequence, men generally feel that they are not needed in antenatal clinics or any other programs that deal with reproductive health, including FP issues. Lack of interest and knowledge on FP issues corroborates a study done by Kaida et al. [45], whose study at Mpigi in Uganda demonstrates that men had limited knowledge about FP. Similar results were obtained in Mbeya region in Tanzania [49]. Likewise, a study in Nigeria by Blanc [58] shows that men had limited knowledge on FP issues. In our study, it was also established that in many situations men were expected to be decision-makers, including on the number of children to have and on whether to use FP methods or not. Generally, this attitude was caused by the patriarchal culture, which puts men in the domineering position and relegates women to subordinate positions. In this study, participants expressed perceptions that men were expected to be the decision-makers because, in many situations, they own the economic resources and, hence, they were the breadwinners.

### Urban–rural differences

Livelihoods in urban areas are generally expensive because everything depends on money, unlike in rural areas where livelihood centers on subsistence farming. People in rural areas get most of their food from their farms thus do not need to buy everything from the shops/markets. In this regard, families in rural areas find it easier to get their food than those urban areas. Also, in urban areas extended families were not as strong as they were in rural areas. Consequently, people in urban areas shouldered the responsibility of taking care of their children as a nuclear family as opposed to the rural areas where children could stay with relatives such as grandparents, aunts and uncles within the extended family set-up.

The promotion of FP services among couples premised on partner involvement with contraception leading to an optimal outcome. The study found that there was more communication among couples in urban areas than among couples in rural and semi-urban areas. Causal factors include association of FP use with infidelity, especially among the females, or lack of adequate information on the importance of using FP as well as cultural factors that limit the discussion of sexual matters especially among rural couples. Perceived association of FP with infidelity in our study corroborates with the findings in Ghana where contraceptive use was commonly associated with perceived infidelity among women [47].

Furthermore, lack of communication among couples in our study corroborates with other studies.

Sujatha and Murthy [56], for example, found that inter-spousal communication on FP was less frequent than communication on other general matters. In this study, it has also been established that there was a dialectical relationship between FP communication and current adoption of FP methods among wives. This is further supported by the significant finding of a higher proportion of users of FP as the communication between husband and wife got better. Other studies done elsewhere came up with similar findings [22-28].

## Conclusion

Study participants have different perceptions of FP methods. Generally, fear of side effects was perceived to be the major hindrance to enhanced use of FP methods. Some participants, especially men, associate the use of FP with unfaithfulness or promiscuity. However, the findings highlight changing gender dynamics among females and males in Mwanza. Indeed, study participants expressed that women are increasingly participating in decision-making around household issues, including number of children and FP method use. As such, family planning programs should encourage communication and joint decision-making among couples in households. Future FP programs should look at addressing underlying social norms leading to gender inequality and lack of joint decision making.

## Competing interest

The authors declare that they have no competing interests.

## Authors’ contributions

IM and RR took part in designing the study, development of tools, data analysis and manuscript writing. DK was involved in writing the manuscript. All the authors approved the final manuscript.

## Authors’ information

IM is a Sociologist and Assistant Lecturer - Muhimbili University of Health and Allied Sciences, Tanzania. Currently a PhD candidate - Radboud University. DK is a Health Psychologist PhD holder; He is a Lecturer - Muhimbili University of Health and Allied Sciences, Tanzania. RR is a Professor. Has a PhD in Developmental Economics and Professor of Economics, Radboud University, The Netherlands.

## Pre-publication history

The pre-publication history for this paper can be accessed here:

http://www.biomedcentral.com/1471-2458/13/523/prepub

## References

[B1] Westoff CF Unmet need at the end of the century. DHS Comparative Reports No. 1. 2001 2001 Calverton, Maryland: ORC Macro

[B2] Bongaarts J Bruce J The causes of unmet need for contraception and social content of services Stud Fam Plann 1995 26 Suppl 2 57 75 7618196

[B3] Campbell M Nuriye NS Malcolm P Barriers to fertility regulation. A review of the literature Stud Fam Plann 2006 37 Suppl 2 87 98 16832983 10.1111/j.1728-4465.2006.00088.x

[B4] Darroch JE Gilda S Haley B Contraceptive technologies: Responding to women’ needs 2011 New York: Guttmacher Institute

[B5] Colombia Demographic and Health Survey Profamilia, Macro International, Inc. Colombia Demographic and Health Survey 2004–2005 2005 Calverton, United States: Macro International, Inc.

[B6] Uganda Bureau of Statistics (UBOS) and ORC Macro Uganda Demographic and Health Survey 2000–2001 2001 Calverton, Maryland, USA: UBOS and ORC Macro

[B7] Sedgh G Rubina H Akinrinola B Susheela S Women with an unmet need for contraception in developing countries and their reasons for not using a method 2007 Occasional Report. New York: Guttmacher Institute

[B8] Bradley S Hilary MS Shane K EK Levels, trends and reasons for contraceptive discontinuation DHS Analytical Studies 2009 20 27 37

[B9] Schuler SR Maria EC Susanna R Misinformation, mistrust, and mistreatment: family planning among Bolivian market women Stud Fam Plann 1994 25 211 221 10.2307/2137904 7985215

[B10] Castle S Factors influencing young Malians reluctance to use hormonal contraceptives Stud Fam Plann 2003 34 3 186 199 10.1111/j.1728-4465.2003.00186.x 14558321

[B11] Rutenberg N Watkins SC The buzz outside the clinics: conversations and contraception in Nyanza Province, Kenya Stud Fam Plann 1997 28 290 307 10.2307/2137860 9431650

[B12] Keesara S Unpublished interviews 2009 Ghana:

[B13] Hall MA Stephenson RB Juvekar S Social and logistical barriers to the use of reversible contraception among women in a rural Indian village J Health Popul Nutr 2008 26 241 250 18686557 PMC2740665

[B14] Plummer ML Wight D Wamoyi JMG Hayes RJ Ross DA Farming with your hoe in a sack; condom attitudes, access and use in rural Tanzania Stud Fam Plann 2006 37 Suppl 1 29 40 16570728 10.1111/j.1728-4465.2006.00081.x

[B15] Tuladhar H Marahatta R Awareness and practice of family planning methods in women attending gyne OPD at Nepal medical college teaching hospital Nepal Med Coll J 2008 10 184 191 19253864

[B16] Obisesan KA Adeyemo AA Fakokunde BO Awareness and use of family planning methods among married women in Ibadan, Nigeria East Afr Med J 1998 75 135 138 9640808

[B17] Nadia DS Campbell M Seema M Misinformation and fear of side-effects of family planning, culture, health & sexuality An Int Res J Intervent Care 2012 14 4 421 433 10.1080/13691058.2012.664659 22390371

[B18] Raine TR Jennifer CG Cherrie BB Sadia H Beth AB Hernandez FA Harper CC Contraceptive decision-making in sexual relationships: young men’s experiences, attitudes and values Cult Health Sex 2010 12 373 386 10.1080/13691050903524769 20169479 PMC2854868

[B19] Bawah A Spousal communication and family planning behavior in navrongo: a longitudinal assessment Stud Fam Plann 2002 33 Suppl 2 185 194 12132638 10.1111/j.1728-4465.2002.00185.x

[B20] Nzioka C Programming for male involvement in reproductive health. Report of the meeting of WHO Regional Advisors in Reproductive Health, WHO/PAHO 2002 Geneva, Switzerland: World Health Organization

[B21] Sharan M Valente T Spousal communication and family planning adoption: effects of a radio drama serial in Nepal Int Fam Plan Perspect 2002 28 Suppl 1 16 25

[B22] Miriam H Kate G Dominick S Brad K Greg G Changes in couples’ communication as a result of a male-involvement family planning intervention J Health Commun 2012 17 Suppl 7 802 819 22545820 10.1080/10810730.2011.650825

[B23] Kang Y O’Donnell C Sparks PL The effect of spousal communication on contraceptive use in Central Terai, Nepal J Patient Educ Couns 2010 81 402 408 10.1016/j.pec.2010.07.018 20719462

[B24] Oyediran K Isiugo-Abanihe U Husband–wife communication and couple’s fertility desires among the Yoruba of Nigeria Afr Popul Stud 2002 17 61 80

[B25] Soldan V How family planning ideas are spread within social groups in rural Malawi Stud Fam Plann 2004 35 275 290 10.1111/j.0039-3665.2004.00031.x 15628785

[B26] Biddlecom A Fapohunda B Covert contraceptive use: prevalence, motivations, and consequences Stud Fam Plann 1998 29 360 372 10.2307/172249 9919630

[B27] Grady WR Klepinger DH Nelson-Wally A Contraceptive characteristics: the perceptions and priorities of men and women Fam Plann Perspect 1999 31 168 175 10.2307/2991589 10435215

[B28] Forste R Morgan J How relationships of U.S. men affect contraceptive use and efforts to prevent sexually transmitted diseases Fam Plann Perspect 1998 30 56 62 10.2307/2991660 9561869

[B29] Frost JJ Darroch JE Factors associated with contraceptive choice and inconsistent method use, United States Perspect Sex Reprod Health 2008 40 94 104 10.1363/4009408 18577142

[B30] Kimuna S Adamchak D Gender relations: husband–wife fertility and family planning decisions in Kenya J Biosoc Sci 2001 33 13 23 10.1017/S002193200100013X 11316391

[B31] Lasee A Becker S Husband–wife communication about family planning and contraceptive use in Kenya Int Fam Plan Perspect 1997 32 15 20

[B32] Odejide T Offering an Alternative to Illegal Abortion in Nigeria New Era Nurs Image Int 1986 2 39 42 1. Calverton, Maryland: ORC Macro 3637707

[B33] Ezeh AC Gender differences in reproductive orientation in Ghana: A new approach to understanding fertility and family planning issues in sub-Saharan Africa Paper presented at the Demographic and Health Surveys: 5–7 1991 Washington, DC: World Conference

[B34] Potts M The unmet need for family planning Sci Am Inc 2000 382 1 88 93

[B35] Greene ME Changing women and avoiding men: gender stereotypes and reproductive health programmes IDS Bull Inst Dev Stud 2000 31 2 49 59 10.1111/j.1759-5436.2000.mp31002007.x

[B36] Gage AJ Women’s socio-economic status, position and contraceptive behaviour in Togo Stud Fam Plann 1995 26 5 264 278 10.2307/2138012 8571441

[B37] Hogan DP Berhanu B Hailemarian A Household organization, women’s autonomy and contraceptive behavior in Southern Ethiopia Stud Fam Plann 1999 30 302 314 10.1111/j.1728-4465.1999.t01-2-.x 10674326

[B38] Mason KO Smith HL Husbands’ Versus wives’ fertility goals and contraceptive use: the influence of gender context in five Asian countries Demography 2000 37 299 311 10.2307/2648043 10953805

[B39] DonaldP M Gender equity in theories of fertility transition Popul Dev Rev 2000 26 427 439 10.1111/j.1728-4457.2000.00427.x

[B40] Dixon-Mueller R The sexuality connection in reproductive health Stud Fam Plann 1993 24 269 282 10.2307/2939221 8296329

[B41] Jacobson JL Transforming family planning programmes: toward a framework for advancing the reproductive rights agenda Reprod Health Matters 2000 8 Suppl 15 21 32 11424265 10.1016/s0968-8080(00)90003-x

[B42] United Republic of Tanzania Tanzania demographic health survey 2010 Dar es Salaam, Tanzania: National Bureau of Statistics (NBS) and ICF Macro

[B43] Dahlgren L Emmelin M Winkvist A Qualitative methodology for international Public Health 2007 Department of Public Health and Clinical Medicine, Umea University

[B44] Graneheim U Lundman B Qualitative content analysis in nursing research: concepts, procedures and measures to achieve trustworthiness Nurse Educ Today 2003 24 Suppl 2 105 112 14769454 10.1016/j.nedt.2003.10.001

[B45] Kaida A Kipp W Hessel P Konde-lule J Male participation in family planning: results from a qualitative study in mpigi district, Uganda J Biosoc Sci 2005 37 269 286 15906884 10.1017/s0021932004007035

[B46] Krakowiak-Redd D Ansong D Otupiri E Tran S Klanderud D Boakye I Dickerson T Crookston B Family planning in a sub- district near Kumasi, Ghana: side effects fears, unintended pregnancies and misuse of a medication as emergency contraception Afr J of Repro Health 2011 15 Suppl 3 121 132 22574499

[B47] Casterine JB Aurora EP Biddlecom A Factors underlying unmet needs for family planning in the Philippines Stud Farm Plann 1997 28 Suppl 3 173 191 9322334

[B48] Fakeye O Babaninyi O Reasons for non use of family planning methods at Ilorin, Nigeria: male opposition and fear of methods Trop Doct 1989 19 Suppl 3 114 117 2773048 10.1177/004947558901900307

[B49] Mwageni EA Ankomah A Powell RA Attitudes of Men towards family planning in Mbeya region, Tanzania: a rural – urban comparison of qualitative data J Biosoc Sci 1998 30 Suppl 3 381 392 9746836 10.1017/s0021932098003812

[B50] Shah NM The role of inter-spousal communication in the adoption of family planning methods Pak Dev Rev 1974 13 454 469 12336153

[B51] Jessica DG Michelle JH ‘Marriage means having children and forming your family, so what is the need of discussion? Communication and negotiation of child bearing preferences among Bangladeshi Couples Cult Health Sex 2007 9 Suppl 2 185 198 17364725 10.1080/13691050601065933

[B52] Speizer IS Are husbands a barrier to women’s family planning use? The case of Morocco Soc Biol 1999 46 1–2 1 16 10842498 10.1080/19485565.1999.9988984

[B53] Miriam H Kate G Dominick S Brad K GG Changes in couples’ communication as a result of a male- involvement in family planning intervention J Health Commun 2012 17 7 802 819 10.1080/10810730.2011.650825 22545820

[B54] Aggleton P Rivers K Scott S Use of the female condom: Gender relations and sexual negotiation. Sex and Youth: Contextual Factors Affecting Risk for HIV/AIDS. A Comparative Analysis of Multi-Site Studies in Developing Countries 1999 Geneva: UNAIDS 104 145

[B55] Khan ME Townsend JW D’Costa S Behind closed doors: a qualitative study of sexual behaviour of married women in Bangladesh Cult Health Sex 2002 4 237 256 10.1080/13691050110102253

[B56] Sujatha DS Murthy MS Husband-wife communication and fertility among two sects of Brahmin J: Fam Welfare 1993 39 Suppl 4 26 30

[B57] Population Council and Interagency Gender Working Group (IGWG) Power in sexual relationships: an opening dialogue among reproductive health professionals 2001 New York

[B58] Blanc AK The effect of power in sexual relationships on sexual and reproductive health: An examination of the evidence Stud Fam Plann 2001 32 189 213 10.1111/j.1728-4465.2001.00189.x 11677692

